# Healing and leishmanicidal activity of *Zanthoxylum rhoifolium* Lam.

**DOI:** 10.3389/fchem.2025.1504998

**Published:** 2025-04-01

**Authors:** Juliana Correa-Barbosa, Heliton Patrick Cordovil Brígido, Bibiana Franzen Matte, Paloma Santos De Campos, Marcelo Lazzaron Lamers, Daniele Ferreira Sodré, Pedro Henrique Costa Nascimento, Gleison Gonçalves Ferreira, Valdicley Vieira Vale, Andrey Moacir do Rosário Marinho, José Edson De Sousa Siqueira, Márlia Regina Coelho-Ferreira, Marta Chagas Monteiro, Maria Fâni Dolabela

**Affiliations:** ^1^ Postgraduate Pharmaceutical Innovation Program, Institute of Health Sciences - Federal University of Pará (UFPA) Belém, Brazil; ^2^ Postgraduate Pharmaceutical Sciences Program, Institute of Health Sciences - Federal University of Pará (UFPA), Belém, Brazil; ^3^ National Council for Scientific and Technological Development (CNPq), Federal University of Pará, Belém, Brazil; ^4^ Biotechnology and Biodiversity Postgraduate Program (BIONORTE), Federal University of Pará, Belém, Brazil; ^5^ Faculty of Dentistry, Institute of Health Sciences - Federal University of Rio Grande do Sul (UFRGS), Porto Alegre, Brazil; ^6^ Faculty of Pharmacy, Institute of Health Sciences - Federal University of Pará (UFPA), Belém, Brazil; ^7^ Postgraduate Program in Chemistry, Institute of Exact and Natural Sciences - Federal University of Pará (UFPA), Belém, Brazil; ^8^ Botany Coordination, Museu Paraense Emílio Goeldi, Ministério da Ciência, Tecnologia, Inovação e Comunicações, Belém, Pará , Brazil; ^9^ Coordinator of the National Institute of Science, Technology and Innovation INCT-PROBIAM Pharmaceuticals Amazonia, Federal University of Pará, Belém, Brazil; ^10^ Postgraduate Neuroscience and Cellular Biology Program, Federal University of Pará, Belém, Brazil; ^11^ Postgraduate Pharmacology and Biochemistry Program, Federal University of Pará, Belém, Brazil

**Keywords:** alkaloids, Rutaceae, *Zanthoxylum rhoifolium*, *Leishmania amazonensis*, cytotoxicity

## Abstract

*Zanthoxylum rhoifolium* is used in folk medicine as an antiparasitic agent. Therefore, this study evaluated the phytochemical aspects and biological activities of *Z. rhoifolium*. For this, the ethanolic extract (EE) was obtained by macerating the peel with ethanol and subjected to acid-base partition to obtain the neutral fractions (FN) and alkaloid fractions (FA). These samples were analyzed using chromatography techniques. From this, a substance was isolated from FN and identified by nuclear magnetic resonance. For biological activity, strains of *Leishmania amazonensis* were used for leishmanicidal activity. For cytotoxicity, cell viability methods were used and finally, the selectivity index (SI) was determined. Cell proliferation assay (SRB method) was also performed, such as a wound healing assay. After analysis, it was inferred that in chromatography, EE, FN and FA presented peaks suggestive of alkaloids, and the alkaloid chelerythrine was isolated from FN. In antiparasitic activity against promastigotes, EE, FN and FA were active. Against amastigotes, the infection inhibition index was dose dependent for EE and FN. In the cytotoxicity test (J774), EE and FN showed moderate cytotoxicity, while FA demonstrated cytotoxicity. In VERO strain, EE and FA showed moderate cytotoxicity, while FN was not cytotoxic. Finally, considering the SI, EE, FN and FA showed high selectivity. Furthermore, EE and FN increased cell proliferation and FN promoted a healing effect. Thus, it is highlighted that the specie *Z. rhoifolium* presented antileishmanial activity and selectivity for the parasite, and its FN presented healing potential.

## 1 Introduction

It is estimated that 350 million people are at risk of contracting leishmaniasis, with approximately 20,000 to 30,000 deaths annually (Paho, 2023). Leishmaniasis is classified according to clinical manifestations, which differ among cutaneous, visceral, and tegumentary manifestations, with the latter being subdivided into mucocutaneous, cutaneous, and diffuse cutaneous manifestations (PAHO, 2023). The species *Leishmania amazonensis*, the causative agent of tegumentary leishmaniasis, can cause wounds that are difficult to heal (Bortoleti et al., 2022). This process involves interactions between cells and various messenger systems, such as cytokines and growth factors, and is divided into 3 phases: inflammatory, proliferative, and remodeling (Ramos et al., 2020).

The treatment of leishmaniases is carried out with pentavalent antimonials (Sb 5+) (Herwaldt, 1999; Rath et al., 2003), amphotericin B, and pentamidines. These drugs are extremely toxic (Mohamed et al., 2023), and there are reports of parasite resistance to these drugs (Domagalska et al., 2023). Most of these drugs are used parenterally, and their treatment is costly. Faced with related problems, the search for therapeutic alternatives has become urgent (Zahra et al., 2023).

Medicinal plants, especially those containing alkaloids, may be promising as leishmanicidal (Veiga et al., 2020; Brígido et al., 2024). *Zanthoxylum rhoifolium* (Rutaceae) is a species that is used as a healing agent and contains alkaloids (Ribeiro et al., 2017; Castillo et al., 2014). An *in vitro* study evaluated the leishmanicidal activity of the ethanolic extract obtained from the bark of *Z. rhoifolium* against macrophages infected with *L. amazonensis* promastigotes, with the best activity occurring at 72h (IC_50_: 9.57 μg/mL) (Melo-Neto et al., 2016). The hydroalcoholic extract of *Z. rhoifolium* leaves showed moderate activity against strains of *L. amazonensis* (IC_50_: 143 μg/mL) (Moura-Costa et al., 2012). The hexane fraction showed *in vitro* activity against macrophages infected with *L. amazonensis* promastigotes and was more promising at 72h (IC_50_: 7.96 μg/mL) (Melo-Neto et al., 2016).

There are still no studies that have evaluated the wound healing activity of this species. However, an *in vivo* study demonstrated that the oil from *Z. bungeanum* seeds increased the proportion of wound healing in a dose-dependent manner and significantly reduced the wound debridement time and the time for complete closure, with the debridement time being shorter than that in the positive control (Li et al., 2017). The authors also revealed a significant increase in the expression of type III collagen protein in wounds (Li et al., 2017). Due to this, the present study describes the antipromastigote and antiamastigote activities and the wound healing potential of ethanolic extracts obtained from *Z. rhoifolium* bark (EE), neutral fractions (FN), and alkaloids (FA). Additionally, the cytotoxicity and possible mechanism of action of the alkaloids isolated from the plant were evaluated.

## 2 Methods

### 2.1 Plant material, processing and chemical studies

The bark of the plant species *Zanthoxylum rhoifolium* was collected and identified by researchers from the Museu Paraense Emílio Goeldi, and its voucher specimen was deposited in the João Murça Herbarium of the same museum, with the identification number MG 224385. The bark powder of *Z. rhoifolium* was subjected to exhaustive maceration with ethanol (ratio of 1:10). The ethanolic solution was filtered and concentrated in a rotary evaporator under reduced pressure until a residue was obtained, resulting in the ethanolic extract (EE) of *Z. rhoifolium*. Fractionation of the EE was carried out to obtain the neutral fraction (FN) and alkaloidal fraction (FA) through acid‒base partitioning.

Mass spectra of the samples were obtained using MAXIS 3G Bruker Daltonics equipment. A reverse-phase C18 column (250 × 4.6 mm) with a 5 µm particle size was used, with UV detection from 200 to 400 nm, a flow rate of 1.0 mL/min, and a column oven temperature of 40°C. The gradient employed consisted of 0.1% formic acid in acetonitrile and 0.1% formic acid in ultrapure water. Mass spectra were obtained using an electrospray ionization (ESI+) system with a capillary voltage of 4,500 and a cone voltage of 500 eV.

The FN were subjected to preparative thin-layer chromatography (TLC) fractionation (dimensions: 0.75 mm) using silica gel as the stationary phase and a dichloromethane:methanol (95:5) mobile phase. The substance was identified using bidimensional methods (COSY and HMBC) and nuclear magnetic resonance spectroscopy (^1^H NMR 400 MHz and ^13^C at 100 MHz).

Chelerythrine: ^1^H NMR data revealed signals characteristic of alkaloids, with the main signals being a *s* at 2.73 ppm, a *s* at 3.92 ppm, a *s* at 3.93 ppm, and a *s* at 6.05 ppm ^13^C NMR detected the presence of 17 signals, most of which refer to the carbons of the aromatic rings (105.6; 149.2; 149.8; 101.4; 139.5; 87.7; 126.4; 153.7; 114.7; 120.2; 121.0; 124.9; 132.8 ppm), however, the presence of other signals stands out at 56.7 ppm (OCH_3_), 62.1 ppm (OCH_3_), 41.1 ppm (CH_3_) and 102.8 ppm (CH_2_ dioxolo). The other 4 carbons in the structure had their displacements defined by HMBC. HMBC: key correlations between H-12 (7.51 ppm) and C-4a (128.0 ppm) and C-10b (123.9 ppm). H-1 (7.15 ppm) with C-4a (128.0 ppm). H-6 (5.55 ppm) with C-6a (126.41 ppm, J2), with C-7 (148.0 ppm), and with the C-5 of the methyl linked to nitrogen (41.13 ppm). H-9 (7.19 ppm) with C-7 (148.0 ppm) and with C-10a (126.3 ppm). The H of the methyl group linked to nitrogen (C-5) at 2.73 ppm, with C-4b (139.55 ppm) with a two-bond distance (J2) and with C-6 (87.67 ppm) with a two-bond distance (J2). The H of the methoxy group (C-7) at 3.92 ppm, with C-7 (148.0 ppm) showing a two-bond distance (J2). The H of the methoxy group (C-8) at 3.93 ppm, with C-8 (153.68 ppm) showing a two-bond distance (J2). COSY: two couplings, one between hydrogen (H-9) and hydrogen (H-10) and the other between hydrogen (H-11) and hydrogen (H-12) (Supplementary Material).

### 2.2 Leishmanicidal activity

#### 2.2.1 Antipromastigote

Strains of *Leishmania* (L.) *amazonensis*, which was isolated from a human patient originating from the municipality of Ulianópolis in the state of Pará, were used and were provided by the Evandro Chagas Institute (IEC, Ananindeua/Pará) under registration number MHOM/BR/2009/M26361. The promastigotes were cultivated in Roswell Park Memorial Institute (RPMI) medium. The test was performed in the logarithmic phase using a suspension of 5 × 10^6^ parasites/100 µL of culture. The samples were tested at doses ranging from 200 to 3,125 μg/mL. As a negative control, culture medium solution and parasite suspension were used. As a positive control, amphotericin B was used. Subsequently, the plate was incubated at 26°C for 24 h. After the incubation period, 10 µL of 3-(4,5-dimethylthiazol-2-yl)-2,5-diphenyltetrazolium bromide (MTT; 5 mg/mL) was added to each well. After 4 h, 10 µL of dimethyl sulfoxide (DMSO) was added, and the optical density (OD) of the samples was measured using a multiplate reader at a wavelength of 490 nm. The percentage of parasites was calculated using the formula adapted from Ngure et al. (2009). Samples were considered very active when the IC_50_ was less than 10 μg/mL (Mota et al., 2015).

#### 2.2.2 Antiamastigote

For the amastigote assay, macrophages of the RAW 264.7 lineage (from the cell bank in Rio de Janeiro) were adhered to circular coverslips (13 mm; 5 × 10^6^ cells) previously inserted into 24-well plates, infected with stationary-phase promastigotes of *L. amazonensis* (2 × 10^6^ parasites), and incubated for 4 h in a 37°C oven with a 5% CO_2_ atmosphere (Silva, 2005). After incubation, the contents of each well were aspirated, and medium containing different concentrations (200 μg/mL, 100 μg/mL, and 50 μg/mL) of EE and FN was added. The negative control consisted of infected macrophages with culture medium without the drug. The positive control consisted of amphotericin B, followed by incubation for 24 h in a 37°C oven with 5% CO_2_. The tests were performed in triplicate. Subsequently, the coverslips were stained with Giemsa stain. The coverslips were observed under a light microscope with a ×40 objective and immersion (×100), where the number of amastigotes per 100 macrophages on each coverslip was determined. The anti-amastigote activity was evaluated using the equation according to Silva (Silva, 2005).

### 2.3 Cell viability and selectivity index

For this assay, MTT and the VERO (from the cell bank in Rio de Janeiro) cell line were used, following the methodology described by Mosmann (1983), and were seeded at 8 × 10^3^ cells/mL. After 24 h, the cells were treated with seven decreasing concentrations of EE, FN, and FA (500 μg/mL to 7.812 μg/mL). After 24 h of treatment, 10 µL of MTT (5 mg/mL) was added. After 4 h, 100 µL of dimethyl sulfoxide (DMSO) was added for complete dissolution of the crystals. The absorbances of the wells were read in a microplate scanning spectrophotometer at a wavelength of 490 nm. The values of the 50% cytotoxic concentration (CC_50_) were calculated using the Galucio (2018) equation. The CC_50_ was determined by linear regression (GraphPad Prism version 6.0 software) and classified as cytotoxic, moderately cytotoxic, or noncytotoxic (Silva-Silva, 2016). The selectivity index was determined by the equation adapted from Reimão (2009). An SI greater than 10 indicates that the compound under study exhibits greater toxicity to the parasite than to the cell line. An SI less than 10 indicates a compound with greater toxicity to the cell line than to the parasite.

### 2.4 Proliferation and wound healing assays

The SRB method was used in HaCaT (from the cell bank in Rio de Janeiro) cells and primary fibroblasts (UFRGS Ethics Committee CAE#59124916.6.0000.5327) at a concentration of 4,000 cells/well. After 24 h, they were treated with different concentrations of EE, FN, and FA (15–700 μg/mL) and then incubated again for 24 h. Subsequently, the cell monolayers were fixed with 10% (w/v) trichloroacetic acid and stained with SRB dye for 30 min. The dye bound to the protein was dissolved in 10 mM Tris base solution for determination of the optical density (OD) at 560 nm using a microplate reader (adapted from Virchai and Kirtikara, 2006).

For the wound healing assay (cell migration), the HaCaT cell line was used. Cells were seeded in 6-well plates and incubated with complete medium at 37°C and 5% CO_2_. When cellular confluence was observed, the monolayer cells were horizontally and vertically scraped with sterile P200 pipette tips to form a cross. Debris was removed by washing with PBS. Cells were treated with EE, FN, or FA at concentrations of 15 or 30 μg/mL. Untreated cells were used as a negative control, and cells treated with solvent (500 μg/mL) were used as a solvent control. The induced scratch representing the wound was photographed at 0h using an inverted phase microscope (Axio Observer Z1, Zeiss, Göttingen, Germany) at ×10 magnification before incubation with treatment. After 12, 24, 36, 48, 60, and 72 h of incubation, new sets of images were taken. To determine the migration rate, the images were analyzed using ImageJ software, and the percentage of the closed area was measured and compared to the value obtained at 0h. An increase in the percentage of closed areas indicated cell migration. Analysis of variance (ANOVA) followed by Tukey’s *post hoc* test was performed to verify differences between groups. The calculation used to determine the % wound closure is represented below:

The % of wound closure was greater than 100 = 12h × 100/0 h.

At 12 h, the percentage of wound closure was greater than 0 = 100.

These calculations were carried out individually for 12, 24, 36, 48, 60 and 72h of treatment.

### 2.5 Molecular docking of the chelerythrine alkaloid

The alkaloid cheleritrina was drawn in MarvinSketch™ and optimized in Avogrado™ to its most stable conformation, applying the MMFF94 force field. The enzymes leishmanolysin (1LML) and trypanothione reductase (TR; 6ER5) were obtained from the public domain RCSB PDB (https://www.rcsb.org/) and optimized using the APBS server (http://server.poissonboltzmann.org/), where charges were added, polar hydrogens were included, and water and cocrystallized solvents were removed from the enzymes.

Molecular docking was performed using the SwissDock server (http://www.swissdock.ch/docking), which utilizes multiobjective scoring constructed from the CHARMM22 force field and the FACTS solvation model, divided into four steps. The server allows the identification of the most favorable clusters through multivariable calculations, which are presented through the discrimination of the binding energy (ΔG) (Haberthür and Cafisch, 2008; Grosdidier et al., 2011). For the enzyme 1LML, which does not contain a cocrystallized inhibitor, a grid box with dimensions of 35 × 35 × 35 (xyz) centered on the Zn in the active site (Mercado-Camargo et al., 2020) was created. For the establishment of the RMSD, a consensus was used where Cheleritrina was subjected to 25 distinct anchorings, and these positions were overlapped, as indicated by the standard deviation. For the enzyme 6ER5, the grid box was established by the inhibitor (2-(diethylamino)ethyl 4-((3-(4-nitrophenyl)-3-oxopropyl)amino)benzoate), from which the following coordinates were extracted: X, 5.986600; Y, −31.853867; and Z, 16.029433. Additionally, redocking was established using the same grid box (Turcano et al., 2018). Amphotericin B was used as the standard drug for docking.

## 3 Results

### 3.1 Chemical studies

**FIGURE 1:**
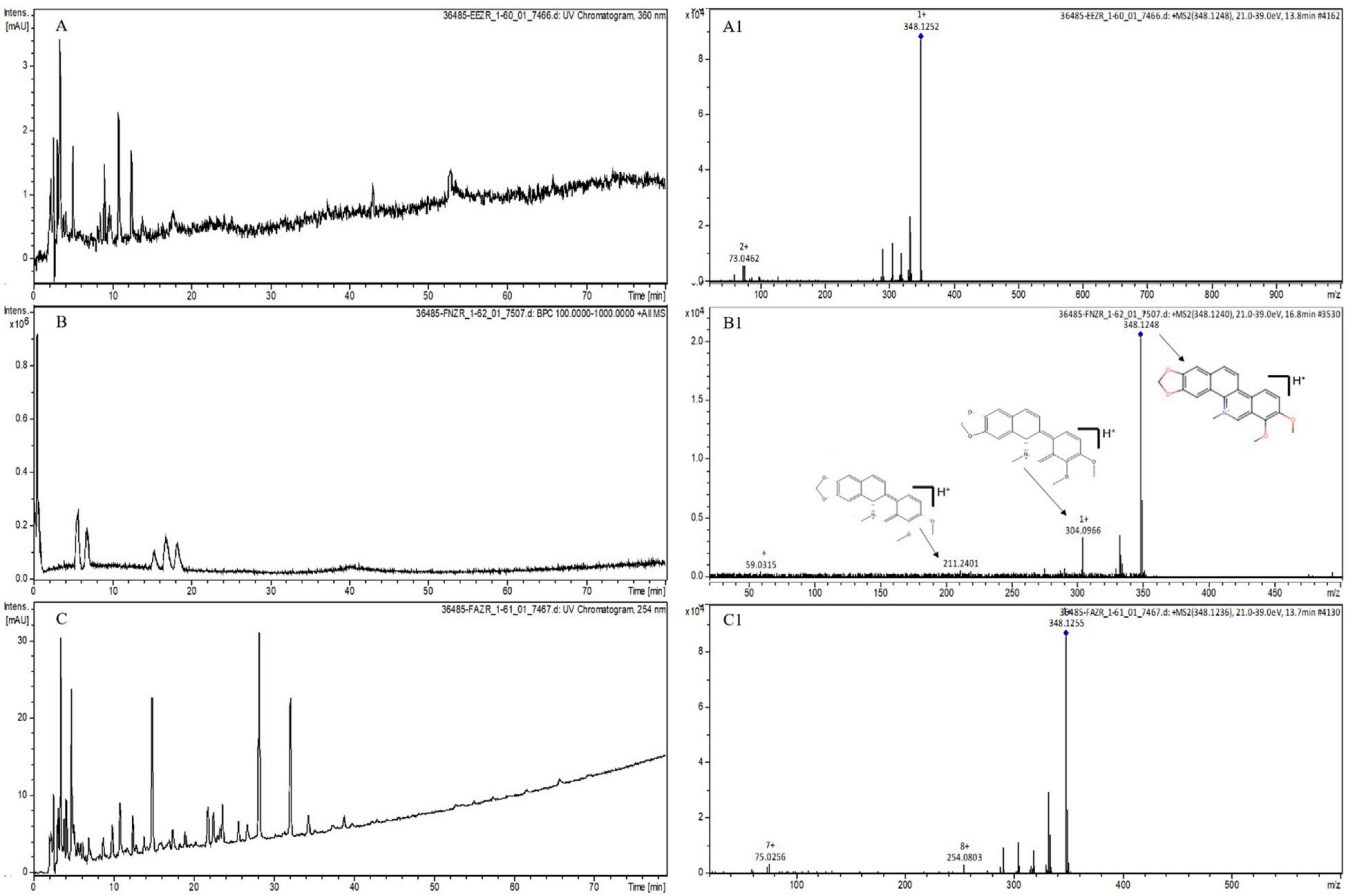
Liquid chromatography of EE, FN and FA of *Zanthoxylum rhoifolium* and suggestive mass spectra of the alkaloid chelerythrine. Conditions: system obtained by an HPLC MS C18 column (5 μm, 250 x 4.6 mm); mobile phase composed of 0.1% formic acid in water **(A)** and 0.1% formic acid in acetonitrile **(B)** O min. 80% A and 20% B, 80 min. 20% A and 80% B; flow rate 1.0 mL/min; temp. 40”C ESI+ ionization voltage with capillary at 4500 eV; cone voltage at 500 eV; reading at a wavelength of 360 nm. Legend: **(A)** Ethanol extract (EE) at 360 nm; **(B)** Neutral fraction (FN) in lull-scan mode; **(C)** Alkaloid fraction (FA) at 254 nm; A1: Mass spectrum for a retention time of 13.8 min in EE; 81: Mass spectrum for a retention time of 16.8 min in FN; C1: Mass spectrum for a retention time of 13.7 min in FA.

**FIGURE 2:**
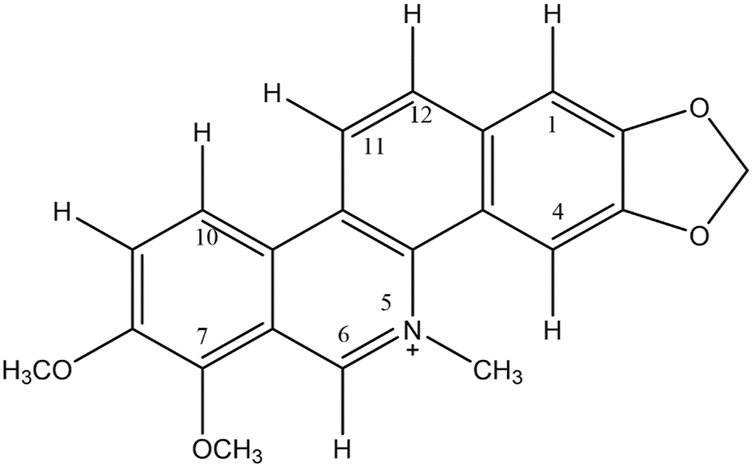
Structure of the benzonanthridine alkaloid chelerythrine, which was isolated from the subtraction obtained from the neutral fraction (FN) of *Zanthoxylum rhoifolium*.

The EE, FN, and FA were subjected to analysis by thin-layer chromatography (TLC) and presented bands suggestive of alkaloids. LC‒MS analyses were carried out to identify the possible alkaloids present in EE, FN, and FA. According to the EE chromatograms and mass spectra, the presence of 9 alkaloids (magnoflorine, laurifoline, magnocurarine, isomagnocurarine, avicine, sanguinarine, chelerythrine, nitidine e oxyavicine) is suggested. Despite differences in retention time (tR) compared to that of EE, similar mass spectra were observed in the chromatogram of FN, and the presence of 7 alkaloids (magnoflorine, laurifoline, avicine, sanguinarine, chelerythrine, nitidine e oxyavicine) previously suggested in EE was suggested. Alkaloids similar to those in EE were found in FA. The presence of alkaloids was suggested by mass spectrometry described in the methodology. Among the alkaloids suggested in the samples, chelerythrine, an alkaloid with a mass of 348 g/mol, was notable (Figure 1). The FN underwent fractionation, and all analyses performed by spectroscopic methods confirmed that the isolated substance was a chelerythrine alkaloid (Figure 2), confirming the mass spectrometry findings.

### 3.2 Leishmanicidal activity and cytotoxicity

**FIGURE 3:**
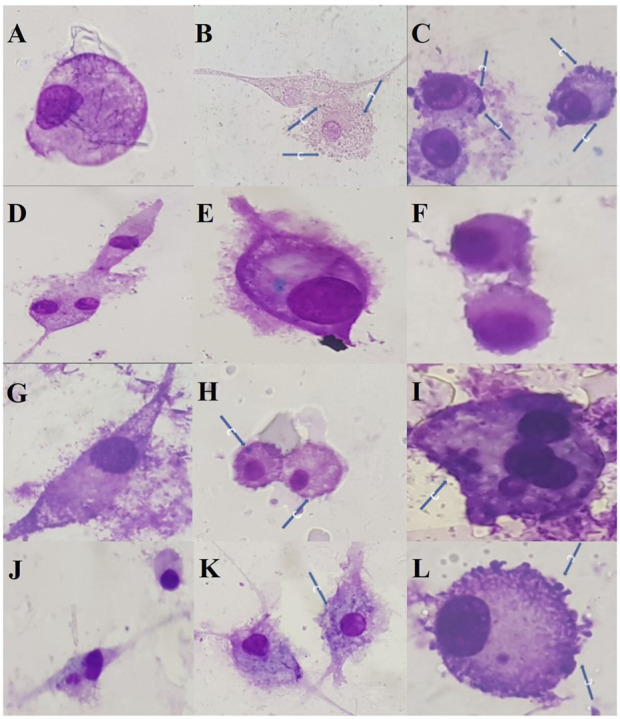
Antiamastigote (*L. amazonensis*) activity of the ethanolic extract (EE) and neutral fraction (FN) obtained from the bark of *Zanthoxylum rhoifolium*. Legend: Reading in MO, 40X magnification: **(A)**: Uninfected macrophage control; **(B)**: Solvent control; **(C)**: Solvent control; **(D)**: Amphotericin B - 10 μg/mL; **(E)**: Amphotericin B - 5 μg/mL; **(F)**: Amphotericin B - 2.5 μg/mL; **(G)**: EE - 200 μg/mL; **(H)**: EE - 100 μg/mL; **(I)**: EE - 50 μg/mL; **(J)**: FN- 200 μg/mL; **(K)**: FN - 100 μg/mL; **(L)**: FN - 50 μg/mL. Arrows indicate the presence of amastigotes.

The specie demonstrated activity against the promastigote forms of *Leishmania amazonensis*, with IC_50_ values of 1.3 μg/mL, 1.0 μg/mL, and 0.9 μg/mL for EE, FN, and FA, respectively (Table 1). Both EE and FA exhibited moderate toxicity to the VERO cell line, while FN showed considerably lower cytotoxic potential (Table 1). Only EE and FN were subjected to investigation of antiamastigote activity, with more significant inhibition indices observed at a concentration of 200 μg/mL, reaching 40% for EE and 50% for FN (Table 2; Figure 3).

**TABLE 1:** Antipromastigote activity, cytotoxicity and selectivity index of Z. *rhoifolium*.

Samples	Antipromastigote		VERO	SI
	IC_50_ (µg/mL) ±SD	Interpretation	IC_50_ (µg/mL)	
EE	1.3 ± 0.2	MA	330.6 ± 0.8	254
FN	1.0 ± 0.4	MA	831.9 ± 0.6	832
FA	0.9 ± 0.2	MA	111.7 ± 0.8	124
Amphotericin B	0.1 ± 0.0	MA	ND	ND

Legend: IC_50_- Concentration Inhibitory 50%; EE, Ethanol Extract; FN, Neutral Fraction; FA, Alkaloid Fraction; VERO, Normal Cells of African Green Monkey Kidney; ND, Not Determined; MA, Very Active; n = 3.

**TABLE 2:** Evaluation of the antiamastigote activity of ethanolic extract (EE) and neutral fraction (FN) in macrophages (RAW 264.7) infected with amastigotes of *Leishmania amazonensis*.

Samples	Inhibition Index (%)/ Concentrations (mg/mL)		
	200	100	50
EE	40 ± 4.3	25.5 ± 2.1	27 ± 7.0
FN	50 ± 5.6	38 ± 1.4	31.7 ± 3.1

	10	5	2.5
Amphotericin B	93 ± 1.5	85 ± 5.0	92.5 ± 2.1

Legend: Ethanolic extract (EE) and Neutral fraction (FN); n = 3.

### 3.3 Proliferative and wound healing effects

**FIGURE 4:**
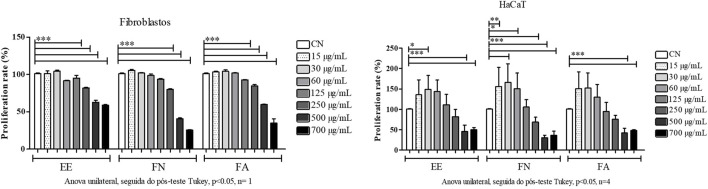
Evaluation of the proliferative effect of Z. *rhoifolium* on keratinocytes (HaCaT) and normal primary fibroblasts. Legend: EE, ethanol extract of *Zanthoxylum rhoifolium*; FN, neutral fraction of Z rhoifolium; FA, alkaloid fraction of Z. rhoifolium . Analysis of variance and Tukey’s post hoc test, p <0.05, n = 1. Asterisks (*) indicate differences between doses.

The proliferative effect of EE, FN, and FA was evaluated on a keratinocyte cell line (HaCaT), where an increase in proliferation was observed at lower concentrations and an inhibitory effect at higher concentrations (Figure 4). Regarding fibroblasts, a similar proliferative effect to that of the control was observed at lower concentrations. However, for the first time pronounced antiproliferative activity was observed, especially at higher concentrations (500 and 700 μg/mL).

**FIGURE 5:**
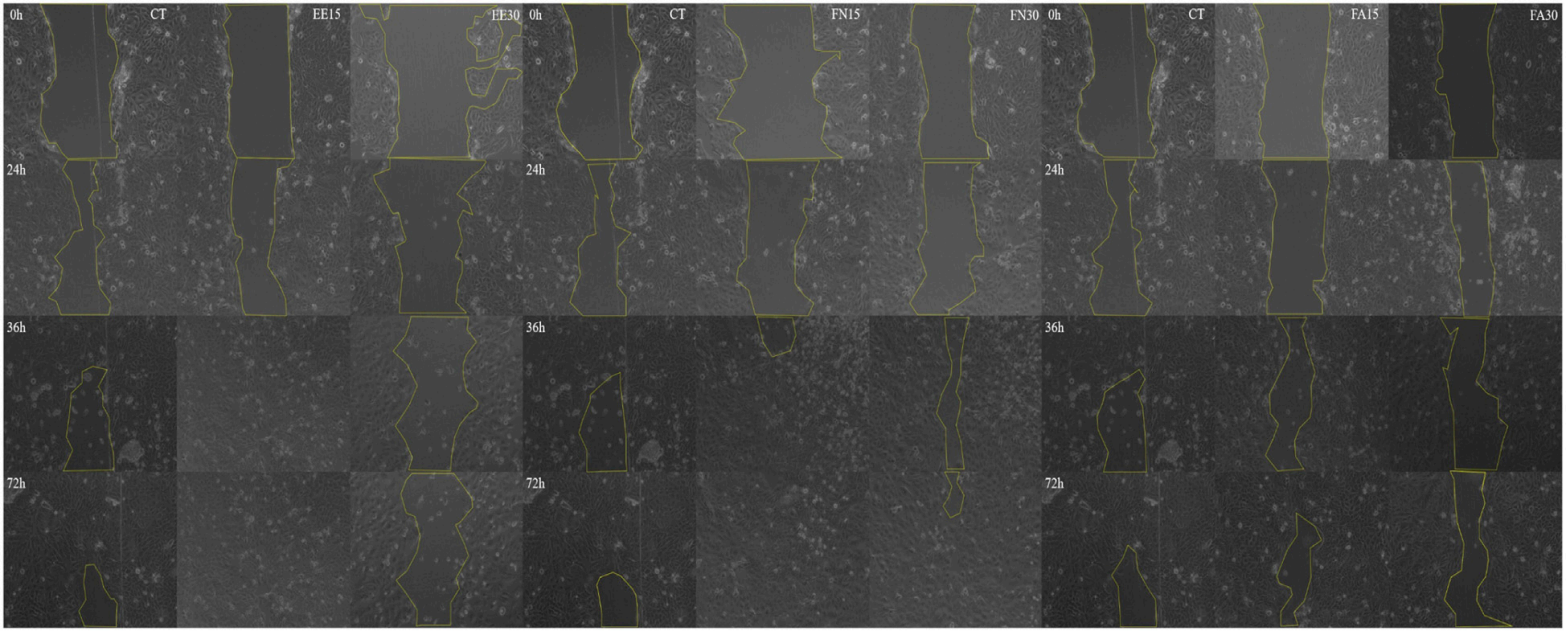
Wound healing assay in *Zanthoxylum rhoifolium* HaCaT cells. Wound closure at 0, 24, 48 and 72 hours. Legend: EE, ethanol extract of *Zanthoxylum rhoifolium*; FN, neutral fraction of *Z. rhoifolium*; FA, alkaloid fraction of *Z. rhoifolium*.

The wound healing potential of EE, FN, and FA was evaluated using a HaCaT cell migration model at 2 concentrations (15 and 30 μg/mL) of each sample, which showed increased proliferation in the previous assay. For EE and FN, wound closure was time dependent, with pronounced closure observed after 36 h of treatment with EE (15 μg/mL) and FN (15 and 30 μg/mL) (Figure 5).

### 3.4 Molecular docking of chelerythrin

**FIGURE 6:**
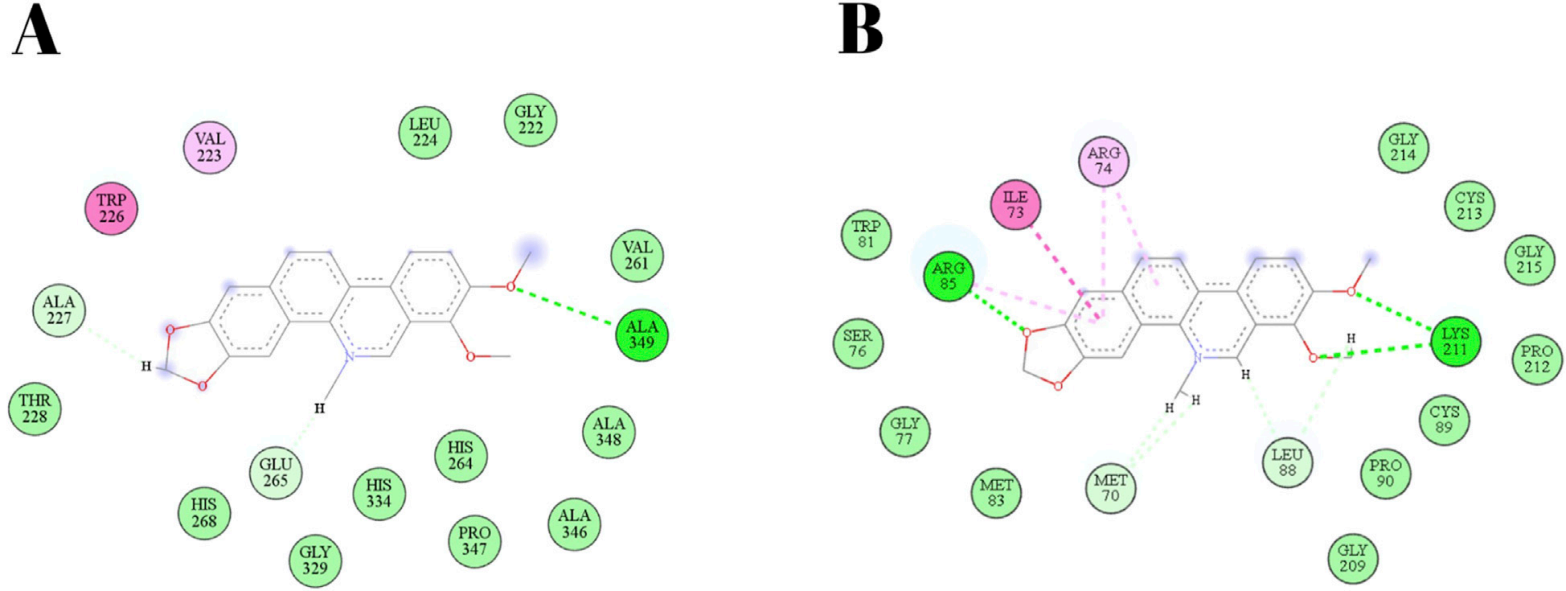
2D diagram of the connections made by cheleritrin. Legend: **(A)** -Leishmanolysin (gp63; 1LML) of *L. major*, **(B)** - trypanothione reductase (TR; 6ER5) of *L.infantum*.

The chelerythrine alkaloid exhibited favorable binding to a leishmanolysin (gp63) with an affinity of −10.17 kcal/mol (total energy: −35.007). Its active site includes the amino acids HIS264, GLU265, HIS268, HIS334, and MET345, along with a zinc atom. Chelerythrine formed two bonds with the amino acid HIS265: one hydrogen bond with a distance of 2.86 Å and one electrostatic bond with a distance of 3.48 Å. Additionally, it formed other hydrogen bonds with ALA227 (2.68 Å) and ALA349 (2.71 Å) and hydrophobic interactions with the residues TRP226 (5.98 Å) and VAL223 (5.10 Å). Upon binding to the pocket, chelerythrine established other interactions, such as van der Waals forces, but did not bind to the other residues belonging to the active site, making it impossible to establish the distance of these interactions. Amphotericin B, used as a control drug, primarily interacted with the GLU220 and GLU265 residues, as well as the zinc atom (Table 3; Figure 6).

**TABLE 3:** Binding affinity (kcal/mol) between chelerythrin and the enzymes leishmanolysin and TR and the types and distance of the bonds made.

Ligands	Binding energy prediction (Kcal/mol)	Number of H-bridges and/or pi-pi interactions; residue.
1LML		
Chelerythrin	-9.14	4; Ala^227^, Ala^349^, Glu^265^
Amphotericin	-11.02	
6ER5		
Chelerythrin	-7.35	8; Arg^85^, Leu^88^, Lys^211^, Met^70^
Amphotericin	-10.37	

When analyzing the coupling of chelerythrine with TR, we obtained a binding affinity of −7.35 kcal/mol (total energy: −13.562), where we observed mainly hydrogen and hydrophobic bonds. The majority of bonds are hydrogen bonds, with bonds to residues ARG85, LEU88, LYS211 and MET70, with distances ranging from 2.22 to 3.09 Å, with chelerythrine forming two bonds with each of these residues. Hydrophobic bonds occur with the ARG74, ARG85 and ILE73 residues (Table 3; Figure 6).

## 4 Discussion

Chemical studies of EE, FN, and FA have demonstrated the presence of alkaloids, including those with indolic, benzophenanthridine, and furoquinoline moieties (Wei et al., 2021; Azonsivo et al., 2023). Additionally, alkaloids such as magnoflorine (Zanon, 2010), avicine, characterized by ^1^H and ^13^C NMR analysis (Jullian et al., 2006; Tavares et al., 2014), chelerythrine, nitidine and oxyavicin were isolated from *Z. rhoifolium*, with structural elucidation by mass spectrometry and ^1^H NMR (Jullian et al., 2006; Zanon, 2010). Furthermore, alkaloids such as laurifoline, magnocurarine and isomagnocurarine were isolated from other species belonging to this genus (Fan et al., 2019) together with sanguinarine, identified by spectrometry mass (Tian et al., 2017).

Studies demonstrate that leishmanicidal activity may be related to the presence of alkaloids (Correa-Barbosa et al., 2023), as example stands out the leishmanicidal efficacy of alkaloid β-carboline (Silva-Silva et al., 2019) and indole alkaloids (Brígido et al., 2020; Veiga et al., 2020). Additionally, previous research has shown that alkaloids isolated from *Z. rhoifolium*, such as avicine and fagaridine, exhibit activity against promastigote forms of *L. amazonensis*, with IC_50_ values below 13.6 µM in both cases. Furthermore, alkaloids from other species of the genus *Zanthoxylum*, such as the alkaloid γ-fagarine isolated from the species *Z. tingoassuiba*, demonstrated activity against *L. amazonensis* promastigotes, with an IC_50_ of 31.3 ± 1.4 µM (Costa et al., 2018).

However, despite not observing a significant interference of fractionation in the antipromastigote activity, a reduction in cytotoxicity was noted, accompanied by an increase in the selectivity index, mainly related to FN. The chemical variation among substances, notably alkaloids, present in the samples may be crucial in explaining the differences observed in cytotoxicity. The fractionation process seems to play a crucial role in attenuating FN toxicity, suggesting a selective process that influences the concentration of less harmful alkaloids. The diversity in the presence of alkaloids can result in distinct cytotoxic responses, as each alkaloid possesses unique properties, and variations in their relative concentrations can directly impact toxicity (Ketema et al., 2023).

FN showed no toxicity; however, EE and FA exhibited moderate cytotoxicity to VERO cells, and this moderate cytotoxicity of EE and FA may be related to the presence of different concentrations of alkaloids, such as sanguinarine, which is known for its cytotoxic properties (Croaker et al., 2022). Another relevant hypothesis is that the presence of a combination of alkaloids in the samples may result in synergistic or antagonistic interactions between these substances. This complexity can influence the total cytotoxic response and explain the differences observed between EE, FA, and FN (Yap et al., 2023). In this regard, the modification of the relative composition of alkaloids in the samples during fractionation is a possible explanation for the alteration in toxicity. The finding that FN has lower cytotoxicity suggests selection during the process, favoring the concentration of alkaloids with noncytotoxic potential.

The antiamastigote effects of EE and FN were tested, and the highest inhibition index was observed at 200 μg/mL. This result is consistent with findings from other studies, where higher concentrations of plant extracts or isolated compounds demonstrated greater efficacy against intracellular parasitic forms (Brigido et al., 2020). The reduction in cytotoxicity and increase in the selectivity index observed for FN, along with its effectiveness against promastigotes and amastigotes, reinforce the promising anti-leishmanial activity of these samples, indicating their potential for application as a source of therapeutic compounds against leishmaniasis.

Given that leishmaniasis causes skin lesions, the proliferative and healing profiles of the most promising samples in this study were examined. Thus, after analyzing the proliferative profile, the results suggested that the higher the tested concentration was, the lower the cell proliferation was compared to that of the control group, indicating that lower concentrations are less cytotoxic. Another study involving cells treated with *Citrullus colocynthis* at concentrations of 100, 50, 25, and 20 μg/mL showed a significant decrease in cell proliferation after 24 h (Yonbawi et al., 2021). However, at lower doses of 10 and 15 μg/mL, it did not reduce the proliferation of HaCaT cells, supporting the presented hypothesis.

An ethnobotanical study conducted in the Brazilian Legal Amazon reported the topical use of decoctions or macerations of the bark or fruits of the species *Z. rhoifolium* for wound healing (Ribeiro et al., 2017), corroborating the findings of the study at lower tested doses. Another study demonstrated that the methanolic extract of *Kigelia africana* showed potential wound healing properties at various concentrations against HaCaT cells, achieving complete wound closure before the control group (Karatay et al., 2023). However, at concentrations of up to 300 μg/mL, it had a low cytotoxic effect, differing from the findings in the present study.

Finally, the observed wound healing activity can be explained by the presence of alkaloids in the samples, as studies have demonstrated the wound healing activity of plants rich in these metabolites, such as *Bowdichia virgilioides* (Barbosa-Filho et al., 2004) and *Solanum xanthocarpum* (Dewangan et al., 2012). However, it is noteworthy that a lesser wound healing effect was observed for FA and at the highest concentration of EE. One hypothesis for the difference in activity could be related to the concentration of these metabolites in each fraction.

Due to the superior activities found in NF, this sample was fractionated until the isolation and identification of the major substance, which was identified as the alkaloid chelerythrine, to which biological activities were attributed. Notably, this metabolite has already shown promising effects when tested alone on strains of *L. amazonensis* amastigotes (Castillo et al., 2014). Thus, to elucidate the possible molecular interactions involved in the mechanism of action of this alkaloid, an *in silico* study was conducted.

Leishmanolysin (gp63) is a metalloprotease of the M8 family that is mainly expressed on the parasite surface (Mercado-Camargo et al., 2020). Studies infer that gp63 affects the parasite’s intracellular survival through the cleavage and/or degradation of fibronectin receptors, which in turn are responsible for a series of functions, including cell adhesion (Maran et al., 1992; Brittingham et al., 1995). Amphotericin B, used as a control drug, mainly interacts with the GLU220 and GLU265 residues and with the zinc atom, and it is known that this drug deregulates the parasite’s membrane. Therefore, we can infer that chelerythrine may disrupt a parasite’s cell wall, leading to its death (Mercado-Camargo et al., 2020).

Thioredoxin reductase (TR) is a fundamental enzyme in parasite survival because it prevents the effects of hydrogen peroxide produced by macrophages, allowing parasite survival and multiplication within the intracellular environment. TR has become an important target because its unique expression in Trypanosomatidae makes this enzyme a safe target for new drugs (Saravanamuthu et al., 2004; Turcano et al., 2018). Although chelerythrine did not bind to the active sites, there was still favorable binding, necessitating additional studies to elucidate this binding. It is possible that chelerythrine may act allosterically, altering the conformation of the target molecule.

Since the alkaloid chelerythrine exhibits high activity against intracellular amastigote forms of *L. amazonensis*, comparable to amphotericin B in terms of the necessary dose for activity, and shows nonspecific toxicity to macrophages, in a model of cutaneous leishmaniasis, it was able to reduce the parasite load by 29% after 6 weeks of treatment (Castillo et al., 2014). These data support our study, which demonstrated that the alkaloid chelerythrine has high leishmanicidal activity, possibly through deregulation of the parasite’s cell wall mediated by inhibition of leishmanolysin (gp63), and contributes to the slight decrease in its survival within macrophages by binding to TR.

## 5 Conclusion

The present study demonstrated that *Z. rhoifolium* is a species with high antileishmanial activity *in vitro*. Furthermore, the species has shown promise in the healing process, due to its ability to increase the rate of cell proliferation. It is worth mentioning that the fractionation of the extract is extremely important, since FN presented the best selectivity index against *L. amazonensis* and was the most promising sample for healing activity. Finally, the need for safety analyzes and *in vivo* biological activities is inferred to confirm the promising potential of the species.

## Data Availability

The raw data supporting the conclusions of this article will be made available by the authors, without undue reservation.
