# Supplementary figures and images for: Healing and leishmanicidal activity of Zanthoxylum rhoifolium Lam
Source: Front Chem. 2025 Apr 1;13:1504998. doi: 10.3389/fchem.2025.1504998 (PMC11996901; doi:10.3389/fchem.2025.1504998)

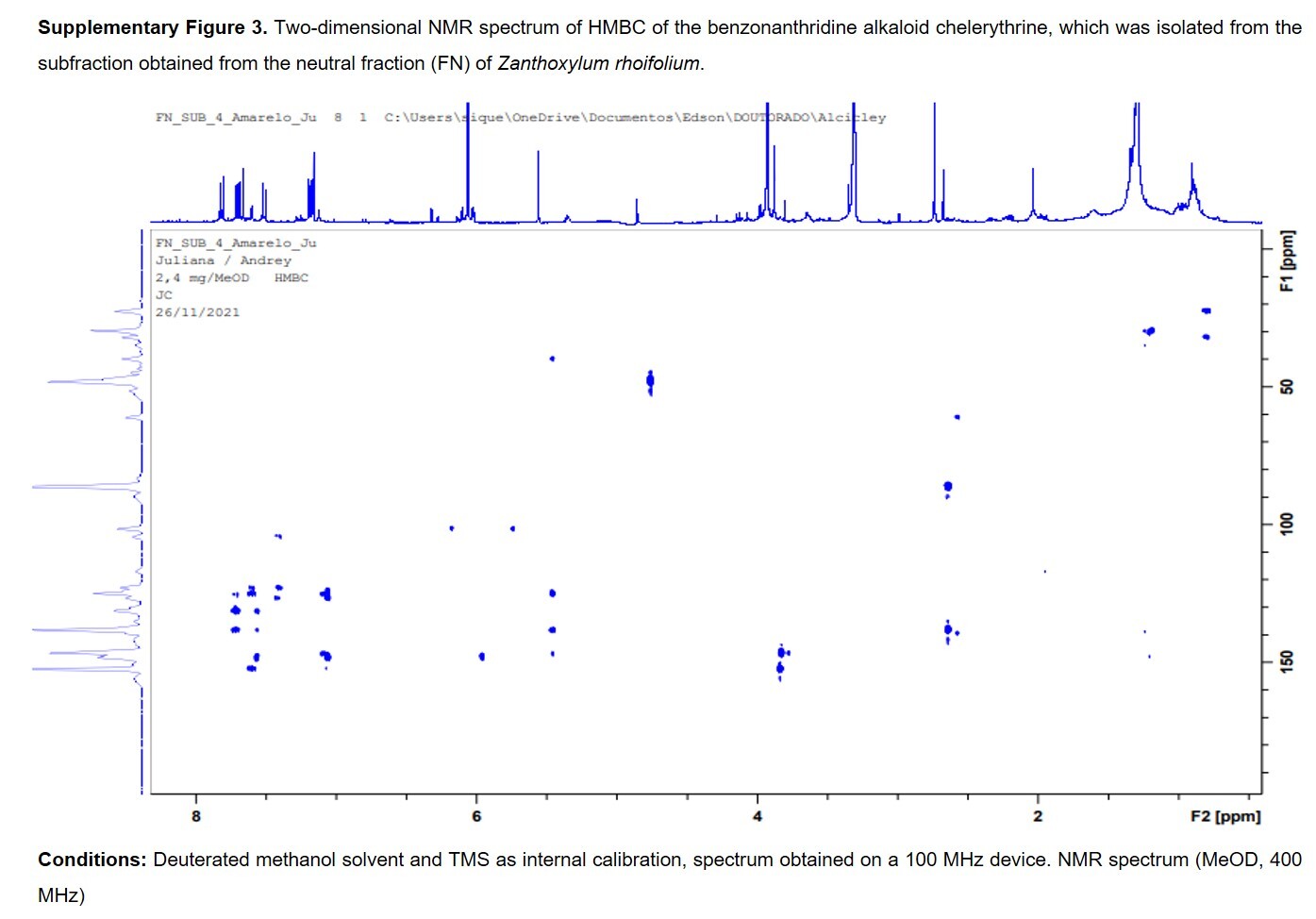

Supplement: Supplementary file 1 [file Image3.jpeg]

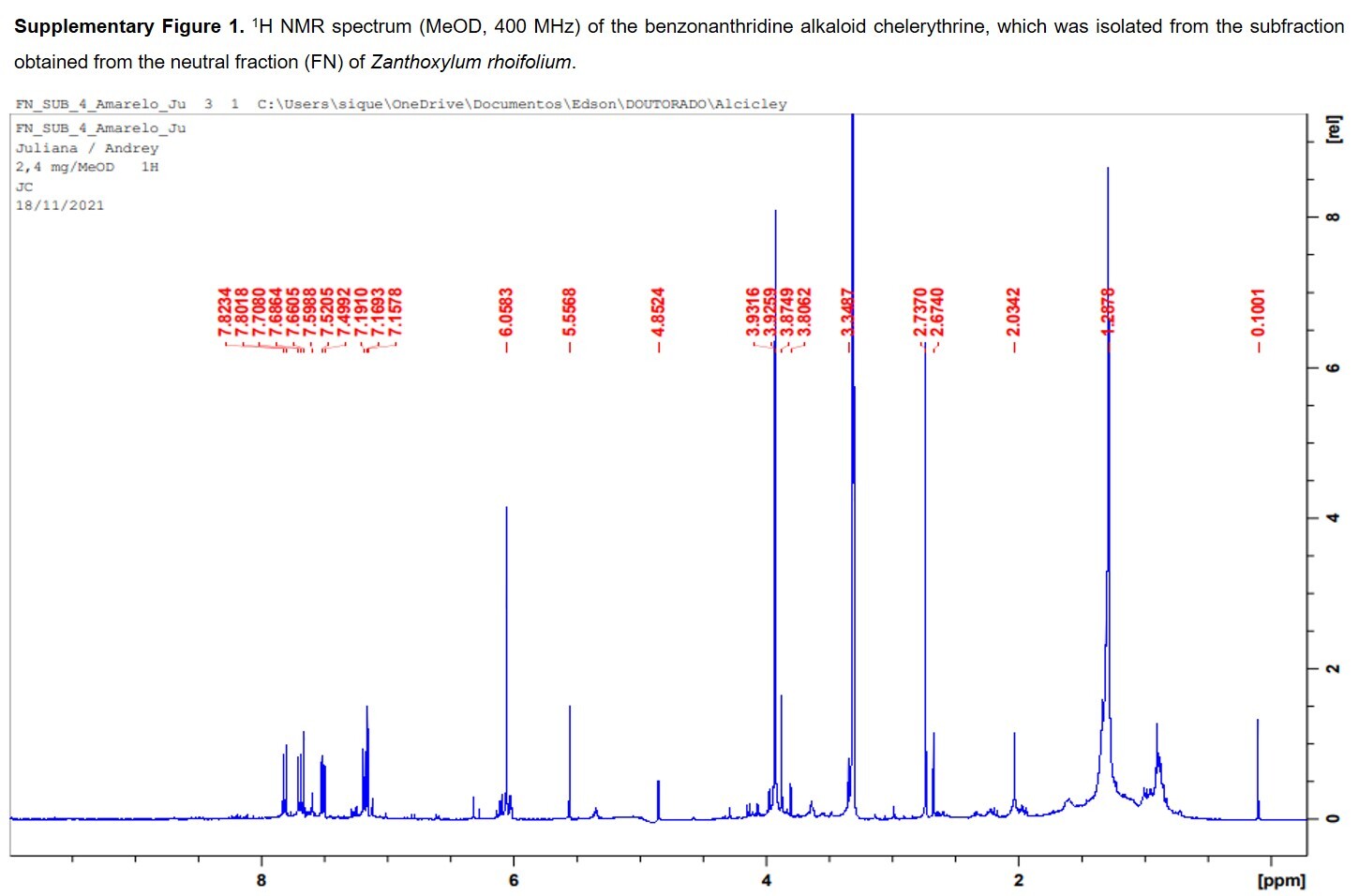

Supplement: Supplementary file 2 [file Image1.jpeg]

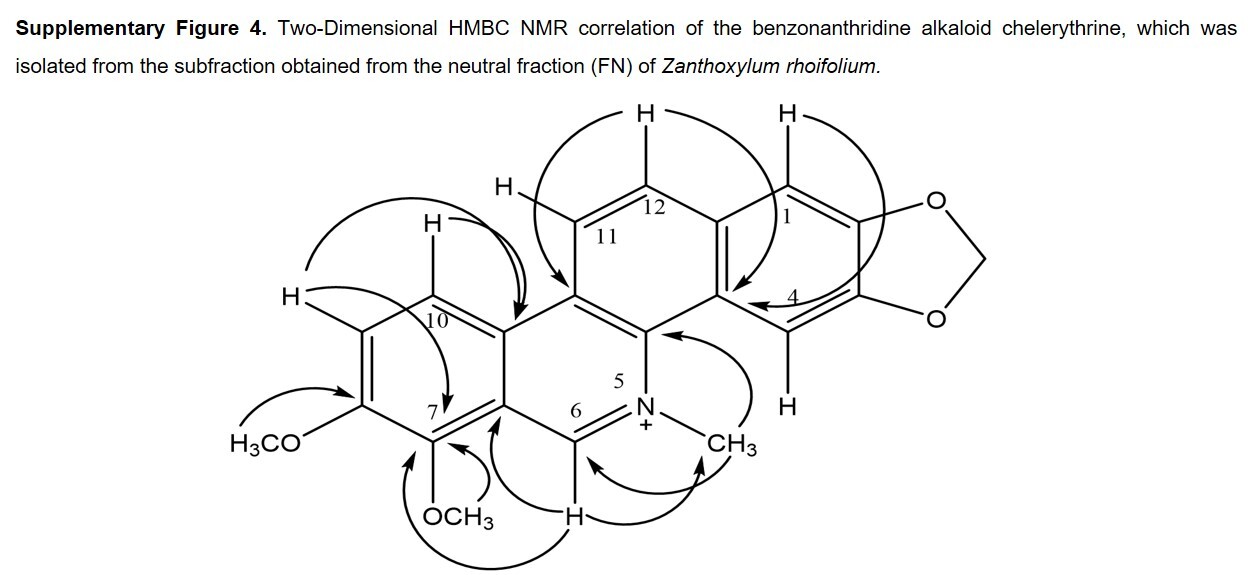

Supplement: Supplementary file 3 [file Image4.jpeg]

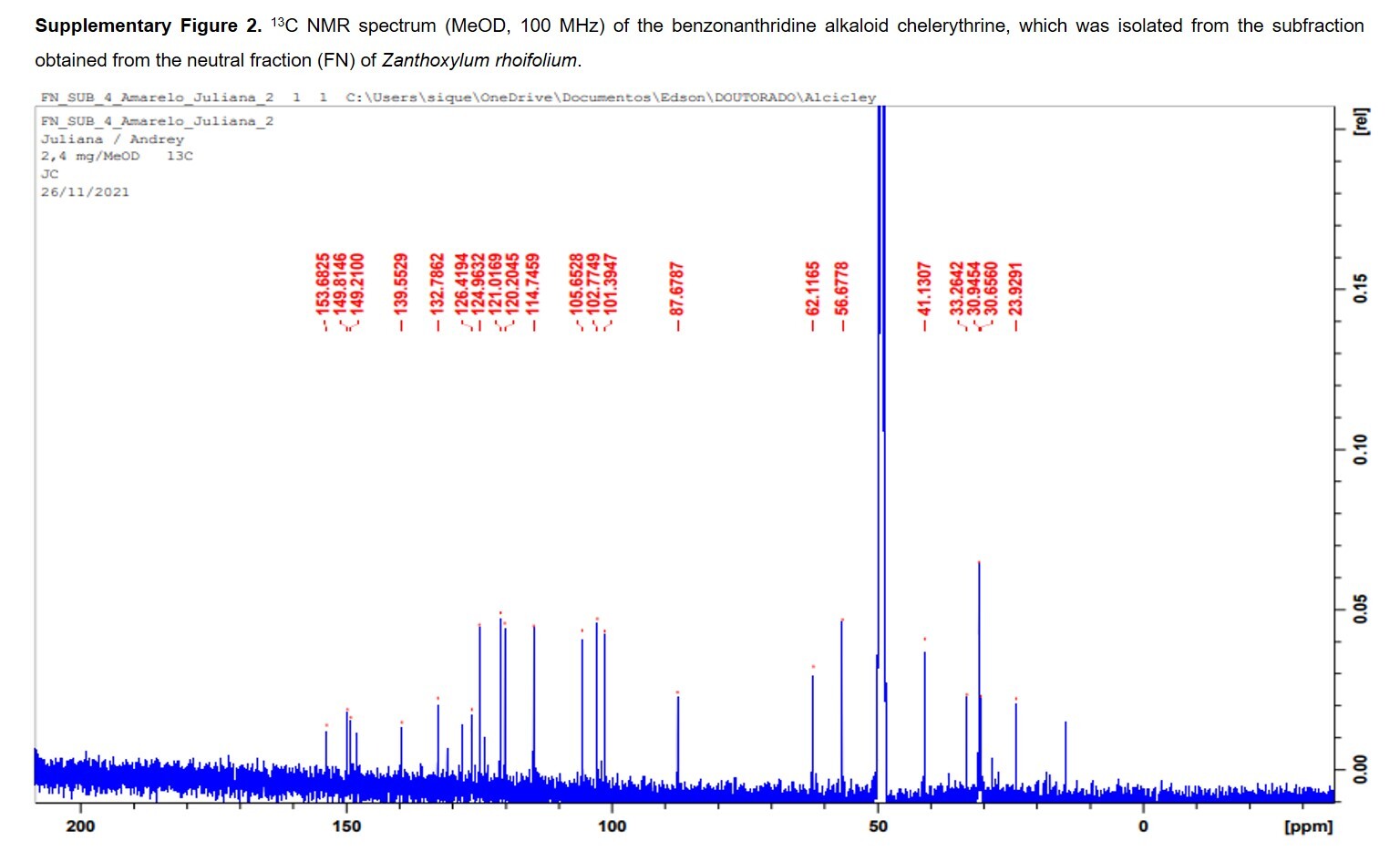

Supplement: Supplementary file 4 [file Image2.jpeg]

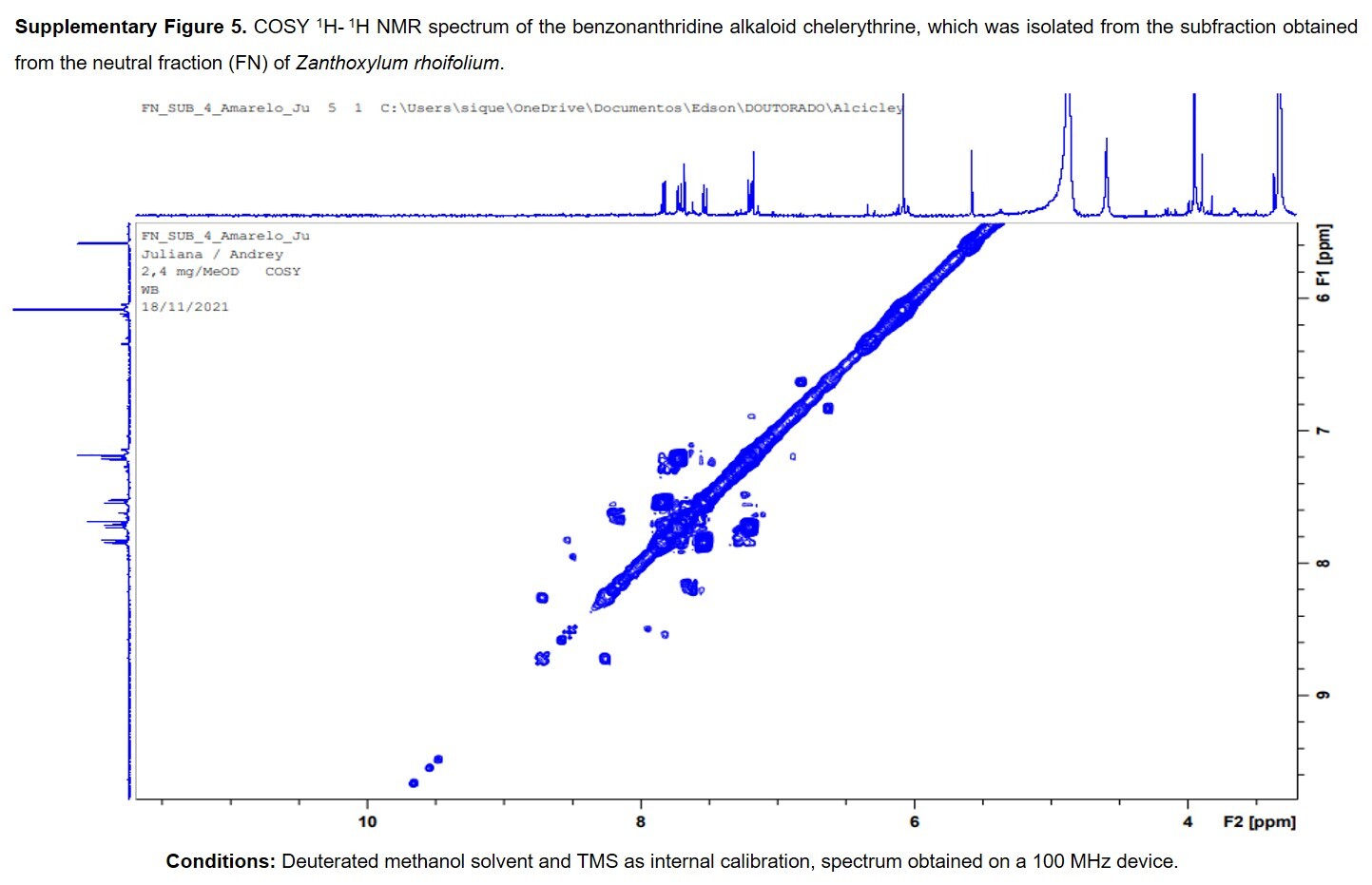

Supplement: Supplementary file 5 [file Image5.jpeg]

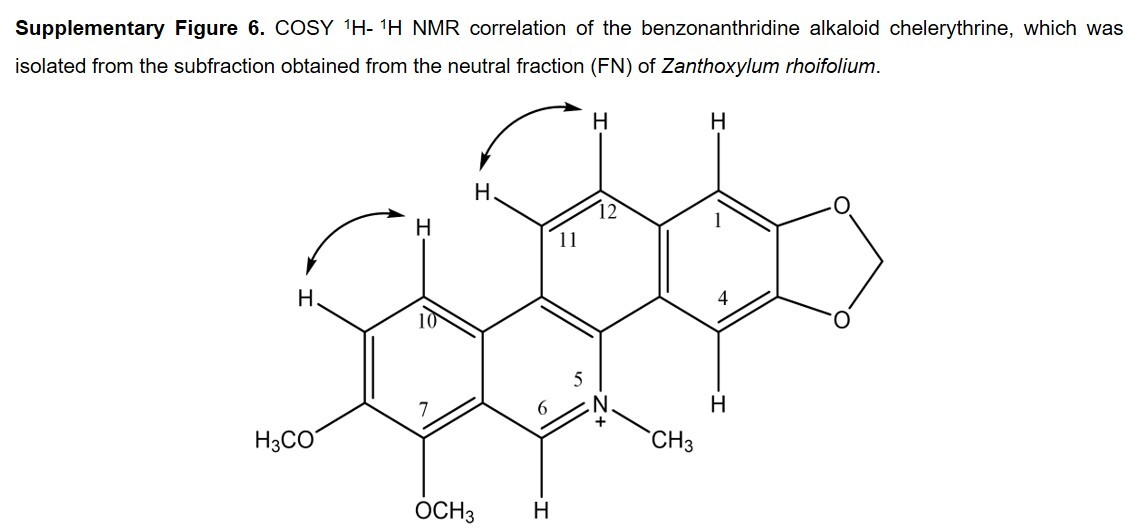

Supplement: Supplementary file 6 [file Image6.jpeg]
